# Froin’s Syndrome Secondary to Traumatic and Infectious Etiology

**DOI:** 10.7759/cureus.6313

**Published:** 2019-12-06

**Authors:** Ashley Garispe, Haaris Naji, Fanglong Dong, Sarkis Arabian, Michael Neeki

**Affiliations:** 1 Emergency Medicine, Arrowhead Regional Medical Center, Colton, USA; 2 Internal Medicine, Arrowhead Regional Medical Center, Colton, USA

**Keywords:** froin’s syndrome, emergency, cerebrospinal fluid

## Abstract

The protein level in the cerebrospinal fluid (CSF) is an important diagnostic tool and, when abnormal, can provide clinicians with clues to the etiology of a patient’s condition. Froin’s syndrome has been described in previous literature as the combination of xanthochromia, elevated protein, and hypercoagulated CSF. The pathophysiology behind Froin’s syndrome is thought to be due to stagnant CSF causing passive and/or active diffusive processes, resulting in hyperproteinosis and hypercoagulation. We present a case of Froin's syndrome in a patient with cervical spine trauma whose extraordinary level of CSF proteinosis helped raise suspicion for underlying obstructive and infectious etiology.

## Introduction

The analysis of the protein level in cerebrospinal fluid (CSF) is an important tool for evaluating a patient's condition. Normal CSF protein levels are usually less than 45 mg/dL. Mild elevations can indicate subtle disruption to the blood-brain barrier and the presence of inflammatory cells and microorganisms [1-3]. Elderly immobile patients, viral encephalitis, and multiple sclerosis are among some disease processes that can contribute to this clinical finding [1-3]. Higher levels of protein, in the ranges of 75-500 mg/dL, can indicate infectious causes with higher degrees of inflammation, such as fungal and bacterial causes, but also non-infectious causes such as hemorrhage, drugs, and myxedema coma. Based on prior literature, levels greater than 500 mg/dL usually indicate defective CSF recirculation and spinal block. This can indicate a spinal obstruction in the forms of abscesses, tumors, degenerative stenosis, or herniations [1-4].

Froin’s syndrome has been described in previous literature as the combination of xanthochromia, elevated protein, and hypercoagulated CSF. This phenomenon was first described by Georges Froin in 1910 upon performing a lumbar puncture on a patient with a known spinal cord tumor [5]. It is postulated to be caused by processes that affect the normal flow of CSF such as tumors or meningeal infections. The pathophysiology is thought to be due to stagnant CSF causing passive and/or active diffusive processes resulting in hyperproteinosis and hypercoagulation [6]. Active tissue, whether malignant or infectious, can accelerate this process, however, cases have been reported where no evidence of infection or malignancy was noted [5]. The prevalence of Froin’s syndrome has yet to be reported.

In this case, we discussed a patient with underlying acquired immunodeficiency syndrome (AIDS) and Varicella zoster virus (VZV) encephalitis who presented to a regional trauma center with signs of acute encephalopathy and evolving fever. The patient was also found to have underlying chronic cervical spinal stenosis exacerbated acutely by trauma. This case illustrates the unique aspect of Froin’s syndrome that is manifested via a combination of acute infectious, traumatic, and chronic degenerative processes.

## Case presentation

A 55-year-old male with an unknown past medical history was transferred by an emergency medical services (EMS) unit as a trauma patient to the closest designated regional trauma center after being involved in a motor vehicle collision.

The patient was found crawling on the ground outside of a vehicle, with major passenger space intrusion that was involved in a collision with multiple other vehicles at freeway speeds. The patient was disoriented and unable to communicate to the EMS team, therefore, it was uncertain if he has been restrained with or if he experienced a loss of consciousness.

Upon initial examination, the patient had a Glasgow Coma Score (GCS) of 10 (Eye 4, Verbal 1, Motor 5) with initial vital signs of blood pressure: 133/81, heart rate (HR): 77, respiratory rate: 18, temperature: 96.8 Fahrenheit (F), and oxygen saturation (SaO2): 91% on room air. He was thin, with significant temporal and extremity muscle wasting. At this time, the trauma team observed no obvious signs of penetrating trauma.

Imaging studies were obtained, which included a computerized tomography (CT) scan of the brain and cervical, thoracic and lumbar spines without contrast. In addition, CT scans of the chest, abdomen, and pelvis with intravenous contrast were performed. Overall, the imaging studies did not reveal any acute traumatic pathology. A trauma laboratory panel, which included complete blood count with differential (CBC with diff), prothrombin time, international normalized ratio, basic metabolic panel, urinalysis, urine drug screen, and blood alcohol level, were also unremarkable.

As the patient remained altered, he was subsequently admitted by the trauma service with concern for possible traumatic brain injury (TBI).

Four hours after the patient’s initial arrival at the trauma center, his mental status began to decline further to a GCS of 8 (Eye 3, Verbal 1, Motor 4). He became increasingly agitated and tachycardic with his HR increasing from the 70s to the high 90s. The patient was subsequently intubated for airway protection. Without overt evidence of trauma and an unremarkable initial workup, the differential diagnosis was broadened to include ischemic, autoimmune, and infectious causes of brain injury, as the trauma scenario presented by EMS seemed unlikely to have caused the patient’s altered mentation.

**Figure 1:**
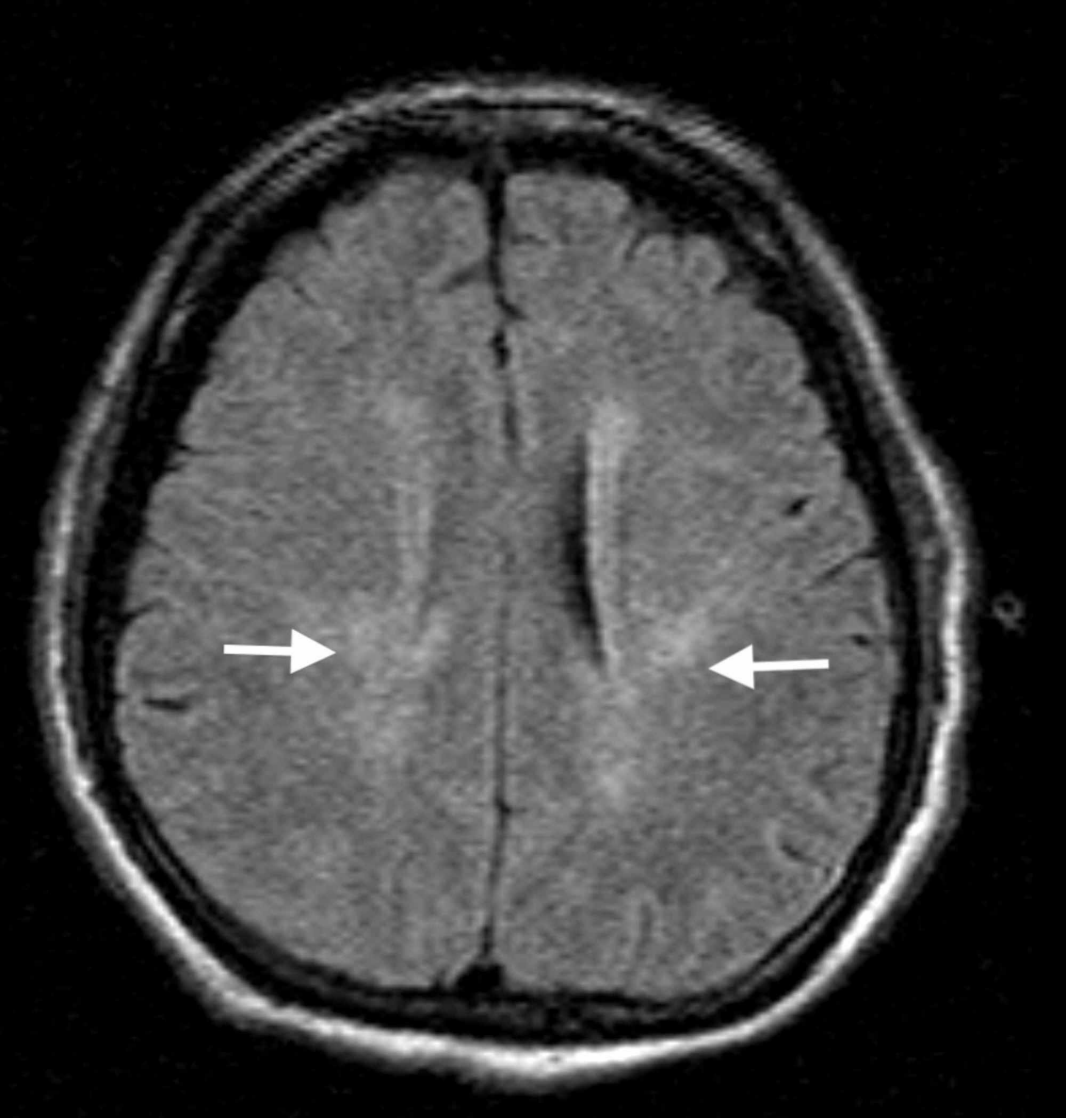
Magnetic resonance imaging of the brain without contrast demonstrating periventricular white matter signal abnormality (white arrows) consistent with chronic ischemic white matter disease versus demyelinating disease.

**Figure 2:**
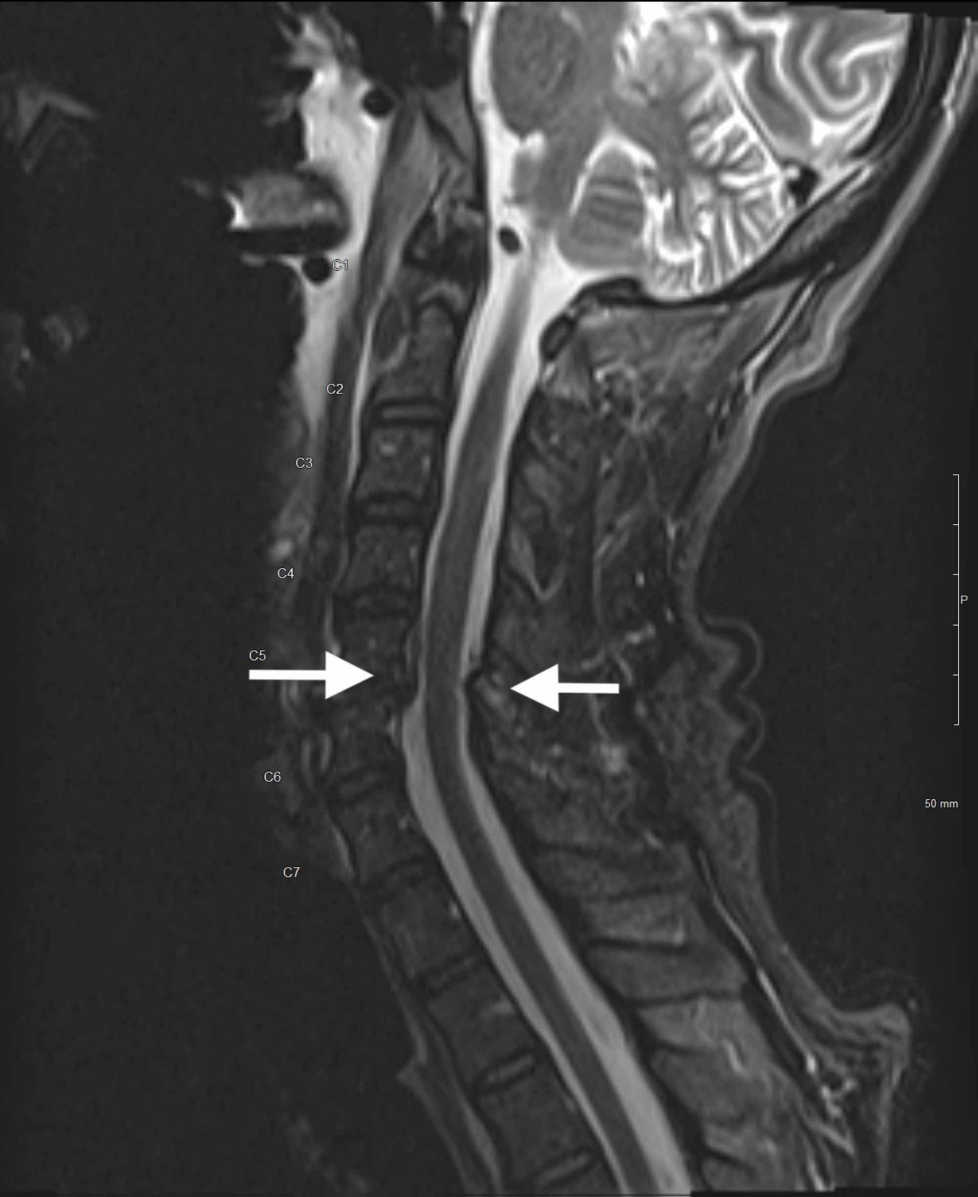
magnetic resonance imaging of the cervical spine without contrast demonstrating cervical stenosis at C5-6 level with prevertebral fluid and edema (white arrows) consistent with Froin’s syndrome.

Because of a high index of suspicion for infectious etiology, the patient was treated empirically with vancomycin, rocephin, decadron, and acyclovir. Additional orders were added, which included thyroid studies, liver function tests, blood/urine/sputum cultures, human immunodeficiency virus (HIV) screening, hepatitis panel, and syphilis screening. Subsequently, the patient underwent CT angiogram of the brain and cervical vessels, magnetic resonance imaging (MRI) of the brain and cervical spine without contrast, as well as a lumbar puncture. Neither angiogram demonstrated any vascular findings. MRI brain demonstrated periventricular white matter signal abnormalities consistent with chronic ischemic white matter disease versus demyelinating disease (Figure 1). MRI cervical spine demonstrated focal disruption of the anterior longitudinal ligament at the C5-6 level with prevertebral fluid/edema causing cervical stenosis (Figure 2).

While CSF results were pending, the decision was made to admit the patient to the medical intensive care unit (MICU) for further management, with the trauma service staying on as a consultant. Shortly after the lateral transfer of care to the MICU, the patient’s HIV screening test resulted positive. The patient also became febrile with his temperature rising to 101.7 F and his HR increasing to 124 bpm. He developed rigors and continued to be agitated despite sedation with a titratable propofol drip.

Upon admission to the MICU, repeat laboratory studies were performed due to the evolving clinical picture. The CBC with diff demonstrated an up-trending white blood cell count (WBC) from 7.8 Th/ul without bands on initial presentation to 11.9 Th/ul with a 17% bandemia. The comprehensive metabolic panel did not demonstrate any severe electrolyte abnormalities. Additionally, cardiac enzymes, thyroid hormone levels, Tylenol and salicylate levels, blood alcohol level, and urine drug screen were all within normal limits.

The patient’s CSF results were as follows: yellow and clear in color with WBC count of 63 (lymphocytes 39%, neutrophils 3%), red blood cell (RBC) count of 78, glucose of 100, and total protein of 1,290 mg/dL.

On hospital day two, the patient’s family was reached and they able to relay that the patient had a two-month, complicated hospitalization for bilateral pneumonia at another facility in a neighboring county occurring four months prior to his current presentation. Those outside medical records demonstrated the patient was HIV positive with a cluster of differentiation 4 (CD4) count of 8 cells/uL. Based on the information obtained from the medical records, the patient was not on a highly active antiretroviral therapy (HAART) regimen at the time of the accident. Due to his worsening clinical picture and a diagnosis of AIDS, the patient’s therapeutic regimen was broadened with the addition of fluconazole, Bactrim, and azithromycin.

On hospital day four, CSF studies indicated the presence of Varicella zoster virus with multiple restriction bands corresponding to the same result obtained from his serum. Thus, the origin of the gammaglobulins could not be differentiated as systemic versus intracerebral. The culture and gram stain of the patient’s CSF were unremarkable. Blood and urine cultures did not demonstrate any growth after five days. Sputum culture grew methicillin-susceptible Staphylococcus aureus (MSSA) and Serratia marcescens.

Additional CSF studies were negative for acid-fast bacillus, Coccidioides antibody, cryptococcus antigen, cytomegalovirus, herpes simplex virus 1 and 2, JC virus, oligoclonal bands, toxoplasma immunoglobulin G (IgG) and immunoglobulin M (IgM) antibodies, West Nile IgG and IgM antibodies, and venereal disease research laboratory test (VDRL).

The patient had a prolonged hospitalization and experienced numerous complications due to his immunocompromised state and natural disease progression. His mentation continued to wax and wane. He developed seizure activity, hyponatremia secondary to cerebral salt wasting, a pleural effusion, aspiration pneumonia, and gastrointestinal bleeding secondary to Kaposi’s sarcoma. 

Late in the hospital course, the patient developed hypoxia and hypotension despite being on maximal oxygen settings, vasopressor therapies, and broad antibiotic coverage. At this point in the hospital course, the family elected for hospice care and comfort measures. The patient expired on day 60 of his hospitalization.

## Discussion

We noted an extraordinary level of CSF proteinosis in this case, one that should raise suspicion for obstruction of CSF flow. In previously reported cases of Froin’s syndrome, several pathological findings affecting the leptomeninges, such as tumors, infections, and degenerative processes, have been described to cause this phenomenon. This patient was presumed to have Varicella encephalitis superimposed with the traumatic exacerbation of cervical stenosis. Table 1 archives prior case reports with Froin’s syndrome and associated CSF findings [4-5,7-9]. It is notable that all the aforementioned causes of Froin’s syndrome have been mentioned in those reports. The significance of these findings is that CSF proteinosis can clue the clinician to evaluate leptomeningeal anatomy further.

**Table 1:** Archived prior case reports with Froin’s syndrome and associated CSF findings (WBC = White blood cells, RBC = Red blood cell, HIV = human immunodeficiency virus, CSF = cerebrospinal fluid)

Case Report	Suspected Cause	WBC Count	RBC Count	Protein level
Govindarajan R et al. [4]	Staph aureus spinal epidural abscess	Within normal limits	888 cells/uL	3295 mg/dL
Kleinschmidt-DeMasters B.K. et al. [5]	Varicella encephalitis in the setting of HIV	1300 cells/uL	4430 cells/uL	1877 mg/dL
Maharjan K. et al. [7]	Tuberculosis/Pott’s disease	Not reported	Not reported	>1500 mg/dL
Kwon, soon-Kul. et al. [8]	Spinal burst fracture/ dislocation	50	none	3114.5 mg/dL
Heckman J.G. et al. [9]	Varicella encephalitis in the setting of Alzheimer's	312 cells/uL	3 cells/uL	625 mg/dL

Previous case series have described an increased signal intensity of CSF that is proximal to an obstruction when compared to distal [10]. Our patient’s cervical MRI did not show this pattern at the level of his cervical spinal stenosis. It would be interesting to reflect on whether our patient’s CSF proteinosis was transient or unrelated to his cervical spinal stenosis and solely due to an inflammatory process from his viral encephalitis. Given the uniform signal intensity of the CSF in our patient’s MRI, we can postulate that the CSF blockage may be at the level of the arachnoid villi [3].

In reported literature, VZV encephalitis is associated with mild pleocytosis and a protein count in the CSF that rarely exceeds 80 mg/dL [11]. There is a case report of Froin’s syndrome in VZV encephalitis that was associated with significant pleocytosis. This case report was associated with CNS vasculitis found on postmortem analysis [1-2]. Our case is the first in the published literature that is associated with isolated CSF proteinosis without significant pleocytosis. Though our patient exhibited radiographic evidence of leukoaraiosis with deep matter changes caused by small vessel disease, we cannot confirm for certain that the patient had a similar presentation.

It is important to consider a broad differential diagnosis for patients who present with altered mental status in the setting of a traumatic injury. Our case emphasizes the importance of maintaining vigilance to track events in the patient’s history that may not be fully discovered in the initial phases of evaluation.

## Conclusions

Froin’s syndrome is a novel medical presentation with a complex pathophysiological process that requires a high index of suspicion and persistent vigilance during the early stages of clinical management.
